# CefaleApp: Una Aplicación Móvil para el Diagnóstico de la Migraña en la Atención Primaria de Salud

**DOI:** 10.31083/RN46506

**Published:** 2026-02-26

**Authors:** Daniel Apolinar García-Estévez, Baltasar García Pérez-Schofield, Nabil Alberto Sabbagh Casado

**Affiliations:** ^1^Servicio de Neurología, Complejo Hospitalario Universitario de Ourense, 32005 Ourense, España; ^2^Grupo de Investigación Neurociencias Clínicas, Instituto de Investigación Sanitaria Galicia-Sur (IIS Galicia Sur), Hospital Álvaro Cunqueiro, 36312 Vigo, España; ^3^Escuela Superior de Ingeniería Informática, Universidad de Vigo, Campus de Ourense, 32004 Ourense, España

**Keywords:** migraña, cefalea tipo tensión, inteligencia artificial, cefalea, atención primaria, salud digital, migraine, tension-type headache, artificial intelligence, headaches, primary care, digital health

## Abstract

**Introducción:**

Las cefaleas son el principal motivo de asistencia en las consultas de Neurología, siendo la migraña la cefalea primaria más frecuente. Nuestro objetivo fue desarrollar una aplicación informática (app) que pudiera empoderar al facultativo de Atención Primaria de Salud (APS) en la toma de decisiones en el campo de la migraña.

**Material y Métodos:**

Se diseñó un sistema de inteligencia artificial basado en reglas que se empleó para tratar las respuestas de los pacientes a las cuestiones que se plantean en el ID-Migraine-screener, y posteriormente determinar si cumplen con los criterios de diagnóstico de migraña o de cefalea tipo tensión de la International Headache Society. La aplicación, que se conoce como *CefaleApp*, está diseñada para generar un diagnóstico de migraña, cefalea tipo tensión o cefalea mixta.

**Resultados:**

*CefaleApp* se validó en 152 pacientes remitidos desde las consultas de APS con la sospecha diagnóstica de migraña o cefalea tipo tensión. Los pacientes se valoraron en la consulta de Neurología de un hospital de segundo nivel y en dos hospitales comarcales. La concordancia en el diagnóstico generado por *CefaleApp* y el emitido por un neurólogo experto en cefaleas (gold-standard), se estimó con el índice Kappa de Cohen, y el coeficiente de correlación de Matthews (CCM). La exactitud diagnóstica global fue del 90,8% (IC 95%: 85,1–94,6%), el índice Kappa fue de 0,73 (IC 95%: 0,59–0,87), y el valor de CCM fue de 0,73.

**Conclusiones:**

El diagnóstico de migraña generado por *CefaleApp* muestra una concordancia sustancial-alta con el emitido por el neurólogo experto en cefaleas.

## Abstract (en)

**Introduction:**

Headaches are the main reason for visits to Neurology clinics, and migraine is the most common primary headache. with migraine being the most frequent. Our objective was to develop a computer application (app) that could empower Primary Health Care (PHC) physicians in decision-making regarding migraine.

**Material and Methods:**

A rule-based artificial intelligence system was designed to process patients’ responses to the ID-Migraine screener and subsequently determine whether they met the diagnostic criteria for migraine or tension-type headache, according to the International Headache Society. The application, known as *CefaleApp*, is designed to generate a diagnosis of migraine, tension-type headache, or mixed headache.

**Results:**

*CefaleApp* was validated in 152 patients referred from PHC clinics with a suspected diagnosis of migraine or tension-type headache. Patients were evaluated in the Neurology Department of a secondary-level hospital and in two regional hospitals. Agreement between the diagnosis generated by *CefaleApp* and that issued by an expert headache neurologist (gold standard) was estimated using Cohen’s Kappa index and the Matthews correlation coefficient (MCC). Overall diagnostic accuracy was 90.8% (95% CI: 85.1–94.6%), Cohen’s Kappa index was 0.73 (95% CI: 0.59–0.87), and the MCC value was 0.73.

**Conclusions:**

The migraine diagnosis generated by *CefaleApp* shows substantial-high agreement with that provided by the expert headache neurologist.

## 1. Introducción

La migraña es una de las enfermedades neurológicas más prevalentes e incapacitantes a nivel mundial. Se estima que afecta a más del 12% de la población, siendo una de las principales causas de discapacidad en adultos jóvenes, especialmente mujeres [1, 2]. El diagnóstico de migraña se puede realizar en la Atención Primaria de Salud (APS) ya que se basa en criterios clínicos bien definidos [3, 4], además se dispone de un test de cribado (ID-Migraine-Screener) de alta sensibilidad y ampliamente validado [5, 6, 7, 8, 9, 10], y es una patología que no precisa de la realización de pruebas complementarias en la mayor parte de los casos [11, 12]. Sin embargo, diversos estudios han constatado que en la APS hay una baja prevalencia de la prescripción del tratamiento sintomático con triptanes y del empleo de los diferentes tratamientos preventivos de la migraña [13, 14, 15, 16, 17, 18]. La transformación digital en salud -con el uso de tecnologías basadas en la inteligencia artificial- permite mejorar la toma de decisiones clínicas, optimizar los recursos sanitarios y empoderar tanto a profesionales como a pacientes [19]. Esta digitalización representa una oportunidad para hacer frente a retos estructurales del sistema de salud como la sobrecarga asistencial, la falta de especialistas o el acceso desigual a servicios de calidad. En este trabajo presentamos una aplicación móvil, denominada *CefaleApp*, que es una herramienta digital diseñada para diagnosticar o confirmar el diagnóstico de migraña y para orientar el tratamiento preventivo de la misma en el primer nivel asistencial, ofreciendo a médicos y pacientes una guía interactiva, accesible y basada en la evidencia clínica [11, 12].

## 2. Pacientes y Métodos

### Desarrollo y Funcionalidades de CefaleApp

**Fig. 1.**
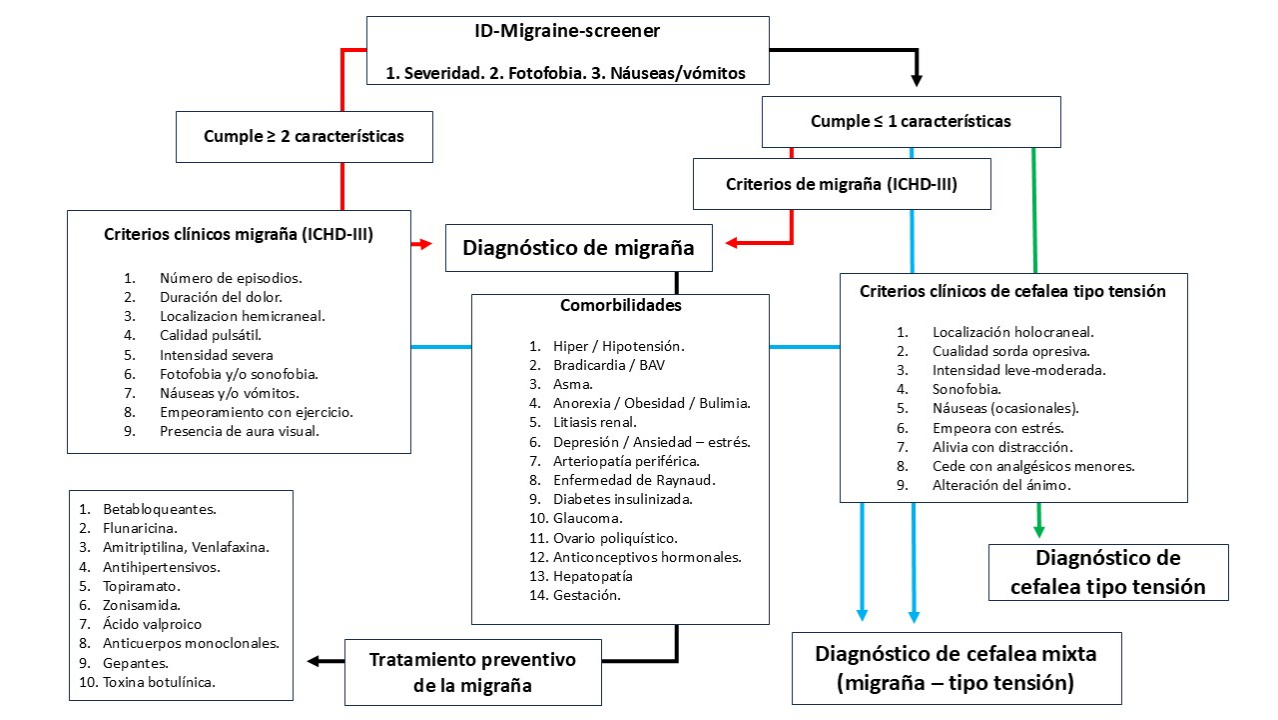
**Algoritmo diagnóstico de migraña y cefalea tensional en el que se basa la programación de *CefaleApp***. Con una puntuación en el ID-Migraine-Screener ≥2, la app orienta las preguntas a diagnosticar una cefalea migrañosa a través de los criterios de la ICHD-III Si la puntuación es ≤1, las preguntas se orientan hacia una cefalea tipo tensión. En los dos supuestos hay posibilidad de que el usuario manifieste la presencia de ambos tipos de cefaleas, generando el diagnóstico de cefalea mixta (migraña- cefalea tipo tensión), e indicando que tipo predomina. Para la migraña, en función de la presencia de comorbilidades, permite orientar el tratamiento preventivo inicial para un caso concreto. BAV, bloqueo aurículo-ventricular; ICHD-III, *International Classification of Headache Disorders*.

Se diseñó un sistema de inteligencia artificial basado en reglas [20, 21] que se empleó para tratar las respuestas de los pacientes a las cuestiones que se plantean en el ID-Migraine- screener que es un test de 3 preguntas probabilísticas de tener clínicamente una migraña [intensidad/discapacidad severa y/o fotofobia y/o náuseas/vómitos]) [5], y posteriormente, determinar si cumplen con los criterios de diagnóstico de la migraña de la *International Classification of Headache Disorders* (ICHD-III) [22], y en caso negativo, la app ofrece la opción de orientar la clínica del paciente hacía la consideración diagnostica de una cefalea tipo tensión (Fig. 1). Por tanto,* CefaleApp* emite el diagnóstico de cefalea tipo migraña siempre que el usuario haya presentado al menos 5 episodios similares de cefalea y la duración de la cefalea sin tratamiento abarque desde 4 horas a 3 días, y en caso contrario emitirá una alerta indicando el incumplimiento de algunos de los criterios A y/o B de la ICHD-III (Tabla 1). Posteriormente, el diagnóstico se basará en la combinación de al menos 2 características del criterio C y al menos 1 característica del criterio D. El criterio E no puede ser evaluado por *CefaleApp* ya que no es una aplicación para el diagnóstico de las cefaleas en general, y es competencia del facultativo de APS descartar una patología subyacente que pudiera ser responsable de una cefalea secundaria, fundamentalmente en base a los resultados de una exploración general y neurológica sin alteraciones, y teniendo en mente las denominadas banderas rojas para la sospecha de la presencia de una cefalea sintomática. En el caso del sexo femenino, las características de una periodicidad de las cefaleas en relación con la menstruación o bien el empeoramiento de la intensidad o frecuencia de la cefalea con la toma de anticonceptivos hormonales constituyen un criterio de apoyo adicional al diagnóstico. En relación al aura, por su complejidad diagnóstica, la aplicación sólo considera el aura visual, ya que está presente en el 90% de los pacientes con diagnóstico de migraña con aura.

**Tabla 1.** **Criterios diagnósticos de la migraña sin aura**.

A	Al menos 5 ataques que cumplan los criterios B-D
B	La cefalea dura entre 4 y 72 horas (sin tratamiento o con tratamiento ineficaz)
C	La cefalea tiene al menos 2 de las siguientes características:
	1. Localización unilateral.
	2. Calidad pulsátil.
	3. Intensidad moderada o severa.
	4. Se agrava por la actividad física rutinaria (caminar, subir escaleras) o el paciente la evita.
D	Durante la cefalea presenta alguno de los siguientes síntomas:
	1. Náuseas y/o vómitos.
	2. Fotofobia y sonofobia.
E	Sin mejor explicación por otro diagnóstico de la ICHD-III.

Este sistema basado en reglas también se empleó para diseñar un algoritmo de selección del tratamiento preventivo basado en las comorbilidades de los pacientes con migraña [23]. El lenguaje de programación para su desarrollo fue Java [24], parametrizando las reglas e información asociada mediante archivos XML (acrónimo de eXtensible Markup Language).

*CefaleApp* está disponible como descarga gratuita para dispositivos Android (URL: https://play.google.com/store/apps/details?id=com.devbaltasarq.cefaleapp ).

Su estructura modular permite un uso flexible, adaptado a distintos perfiles clínicos y situaciones asistenciales. *CefaleApp* está diseñada para diagnosticar/confirmar la sospecha diagnóstica de migraña o de cefalea tipo tensión, no para el diagnóstico de las cefaleas en general, y las únicas sugerencias diagnósticas que genera son de migraña, de cefalea tipo tensión o de cefalea mixta (indicando el predominio de migraña o tipo tensión). Entre sus principales funcionalidades dispone de:

(a) Un algoritmo de diagnóstico basado en los criterios diagnósticos de la Clasificación Internacional de las Cefaleas [22] (Fig. 1).

(b) Un algoritmo de tratamiento, sintomático y preventivo, que tiene en cuenta las comorbilidades del paciente, e incluye las situaciones especiales de la gestación y de la lactancia, ayudando al profesional a seleccionar el tratamiento más adecuado.

(c) Un mini-vademécum específico de los fármacos más utilizados en migraña, incluyendo información sucinta sobre posología, efectos adversos y contraindicaciones.

(d) Escalas de medición del impacto y discapacidad que genera la migraña en los pacientes (MIDAS: Migraine Disability Assessment Scale; HIT-6: Headache Impact Test-6) para el seguimiento clínico evolutivo,

(e) Un apartado de educación sanitaria con preguntas frecuentes y explicaciones comprensibles para el paciente (empoderamiento del paciente) y personal sanitario no especializado (formación continuada).

## 3. Pacientes

Los pacientes que participaron en la evaluación de la concordancia y de la validez de *CefaleApp*, fueron derivados a las consultas de Neurología desde la APS con la sospecha diagnóstica de migraña o de cefalea tipo tensión, bien para confirmar el diagnóstico o bien para iniciar un tratamiento sintomático y/o preventivo. Los pacientes se evaluaron en el ámbito hospitalario, tanto en un hospital de segundo nivel asistencial como en dos hospitales comarcales, en consultas de neurología general, y por un único neurólogo con especial dedicación a la migraña. Los pacientes se incluyeron de forma consecutiva según su programación en la consulta ambulatoria. En primer lugar, se les solicitaba a los pacientes si querían cumplimentar la app, y en caso afirmativo, y tras dar su consentimiento, se les proporcionaba un teléfono móvil con la aplicación *CefaleApp*. Esta app es de auto cumplimentación por el paciente y se realiza en un tiempo medio inferior a 2 minutos, y tras la emisión del diagnóstico clínico por el neurólogo, se comprobaba el diagnóstico generado por *CefaleApp*, procediendo a la inclusión en un registro el motivo de la derivación realizada desde la APS, el diagnóstico emitido por el neurólogo y el generado por *CefaleApp*.

Este proyecto es un estudio observacional de validación, con inclusión consecutiva de pacientes. El desarrollo y validación de *CefaleApp* dispone del informe favorable del Comité de Ética de la Investigación con medicamentos de Galicia con el código de registro 2024/528. Los pacientes que participaron en la validación de la app dieron su consentimiento informado por escrito.

### Análisis Estadístico

Se calculó un tamaño de muestra para cuantificar el grado de acuerdo entre *CefaleApp* y un neurólogo clínico, usando la fórmula para estimar tamaños de muestra en estudios de concordancia con un nivel de confianza del 95%. Para dicha estimación, se ha considerado un coeficiente Kappa esperado de 0,85 (grado de acuerdo casi perfecto). Así mismo, una proporción de clasificaciones positivas por el neurólogo del 80% y una proporción de clasificaciones positivas por *CefaleApp* del 70%. Con estos datos y una precisión (error permitido) del 5%, se ha encontrado que será necesario incluir a 152 pacientes que acudan a la consulta de neurología por dolor de cabeza. Se calculó el índice Kappa que es una medida de concordancia que se utiliza para evaluar el rendimiento de un clasificador. Este índice tiene un rango de valores que va desde 0 (nula concordancia) hasta 1 (concordancia perfecta). Valores de Kappa 0,60–0,80 se consideran de un acuerdo sustancial-alto, y los valores >0,80 son indicativos de una muy buena concordancia. Los análisis estadísticos se llevarán a cabo con el programa *Statistical Package for Social Sciencies* (SPSS) versión 29,0 (IBM Corporation, Armonk, NY, USA).

Además de las métricas clásicas empleadas en estudios de validación diagnóstica (sensibilidad, especificidad, valores predictivos y exactitud), incluimos también un conjunto de métricas avanzadas de rendimiento clasificatorio, habitualmente utilizadas en el ámbito de la bioinformática y el aprendizaje automático. Estas métricas permiten una evaluación más completa del equilibrio entre falsos positivos y falsos negativos, y ofrecen una medida más robusta de la capacidad discriminativa global de la herramienta.

En concreto, se calcularon:

F1-score, utilizado en las ciencias de la computación, es un índice que integra precisión y sensibilidad en un único índice. Es especialmente útil en contextos donde el desequilibrio entre clases podría sesgar la interpretación de la exactitud (en nuestro caso, más diagnósticos de migraña que de no migraña). F1-score es la media armónica entre la precisión (VPP) y la sensibilidad (recall): F1 = 2 ⋅ (Precisión × Sensibilidad) / (Precisión + Sensibilidad). Tiene en cuenta tanto los falsos negativos como los falsos positivos. En nuestro caso un valor de F1 cercano a 1 significa que la app detectaría correctamente casi todas las migrañas (alta sensibilidad) y, además, cuando predice migraña suele acertar (alta precisión).

*Matthews Correlation Coefficient* (MCC), procede del ámbito de la bioinformática y de las ciencias de la computación, considerado una de las métricas más equilibradas y robustas para evaluar clasificadores binarios, ya que tiene en cuenta todos los elementos de la matriz de confusión (VP, VN, FP, FN). El rango de valores oscila entre –1 a +1 (+1 = clasificación perfecta, 0 = clasificación al azar, –1= clasificación completamente errónea).

Índice de Youden (J), procede del ámbito clínico-epidemiológico, y se emplea en la investigación clínica como medida sintética de rendimiento diagnóstico, que combina sensibilidad y especificidad en un único parámetro. Se calcula con la fórmula: J = Sensibilidad + Especificidad – 1. El rango de valores es de 0 a 1 (0 = sin discriminación, 1= perfecto). Se interpreta como la capacidad global de discriminación del test.

## 4. Resultados

Se evaluaron 152 pacientes. El 74,3% fueron mujeres (n = 113). La mediana de edad fue de 44 años (rango: 15–82). La distribución de pacientes por edad fue: <30 años: n = 33 (21,7%), entre 30–44 años: n = 58 (38,2%), entre 45–59 años: n = 38 (25,0%), y ≥60 años: n = 23 (15,1%). Se construyó una tabla de contingencia 3 × 3 con los diagnósticos del neurólogo y de *CefaleApp* para las categorías diagnósticas de migraña, cefalea tipo tensión o cefalea mixta (Tabla 2).

**Tabla 2.** **Tabla de contingencia 3 × 3**.

	*CefaleApp* (migraña)	*CefaleApp* (cefalea tipo tensión)	C*efaleApp* (cefalea mixta)	Total
Neurólogo (migraña)	115	4	0	119
Neurólogo (cefalea tipo tensión)	4	16	1	21
Neurólogo (cefalea mixta)	5	0	7	12

Las medidas globales indican una exactitud diagnóstica del 90,8% (IC 95%: 85,1–94,6%), un índice Kappa de 0,73 (IC 95%: 0,59–0,87), y un valor de MCC de 0,73. En la Tabla 3. se muestran los índices de la validación para las categorías de migraña, cefalea tipo tensión y cefalea mixta.

**Tabla 3.** **Medidas de rendimiento y concordancia entre *CefaleApp* y el diagnóstico del neurólogo experto en cefaleas**.

	Sensibilidad (IC 95%)	Especificidad (IC 95%)	VPP (IC 95%)	VPN (IC 95%)	F1-score	Indice de Youden
Migraña	96,6% (91,6–99,1)	72,7% (57,5–84,6)	92,7% (86,7–96,6)	85,7% (69,7–95,2)	0,95	0,71
Cefalea tipo tensión	76,2% (52,8–91,8)	97,0% (92,2–99,2)	80,0% (55,2–94,7)	96,2% (91,0–98,9)	0,78	0,73
Cefalea mixta	58,3% (27,7–84,8)	99,3% (96,4–100,0)	87,5% (47,4–99,7)	96,5% (92,0–99,0)	0,70	0,58

VPP, Valor predictivo positivo; VPN, Valor predictivo negativo.

En el análisis por subgrupos diagnósticos, la aplicación alcanzó un rendimiento excelente para migraña, con una sensibilidad del 96,6% y un valor predictivo positivo del 92,7%. El F1-score fue de 0,95. En el caso de la cefalea tipo tensión, la app mostró un rendimiento también satisfactorio, con una sensibilidad del 76,2% y una especificidad del 97,0%, aunque con un ligero descenso del F1-score (0,78). La categoría mixta fue la más difícil de identificar, con una sensibilidad moderada (58,3%) pero una especificidad muy elevada (99,3%), lo que implica que, aunque *CefaleApp* detecta menos casos mixtos, rara vez clasifica erróneamente a otros pacientes como tales. En esta categoría, el 41,7% de los pacientes que fueron clasificados como pacientes con migraña por *CefaleApp*, fueron clasificados como cefalea mixta “de predominio migraña” por el neurólogo.

## 5. Discusión

*CefaleApp* representa una innovación digital aplicada a la práctica clínica real. Su diseño está alineado con los principios de la medicina digital: (1) apoyo a la decisión clínica de diagnóstico y tratamiento, (2) integración de escalas validadas y datos cuantificables, (3) uso de algoritmos estructurados con lógica condicional, y (4) interoperabilidad potencial con otros sistemas y plataformas.

La inclusión en la validación de métricas avanzadas como son el MCC, el F1-score, y el índice de Youden, aporta un marco más sólido para interpretar la validez de *CefaleApp* y facilita la comparación con estudios recientes en el ámbito de la salud digital. En la Tabla 4 se muestra una comparativa de las medidas de rendimiento y concordancia entre *CefaleApp* y diversos modelos digitales de diagnóstico de migraña previamente publicados [25, 26, 27, 28, 29].

**Tabla 4.** **Comparativa de las medidas de rendimiento y concordancia en diferentes modelos digitales de diagnóstico de migraña**.

	Tipo de estudio	Exactitud (%) (IC 95%)	Sensibilidad (%) (IC 95%)	Especificidad (%) (IC 95%)	Kappa de Cohen (IC 95%)	Matthews Correlation Coefficient	Índice de Youden
*CefaleApp* (España, 2025)	App vs consulta presencial	90,8 (85,1–94,6)	96,6 (92,6–99,1)	72,7 (57,5–84,6)	0,73 (0,59–0,87)	0,73	0,71
Cowan RP *et al*. (EEUU, 2022) [25]	Cuestionario online vs entrevista telefónica	92 (87–95)	89 (87–95)	97 (90–100)	0,83 (0,75–0,91)		
Kim KM *et al*. (Corea, 2022) [26]	Cuestionario online vs entrevista telefónica	93,8 (90,1–96,4)	92,6 (84,6–96,5)	94,8 (89,6–97.1)	0,87 (0,81–0,93)		
Han X *et al*. (China, 2023) [27]	Registro informático vs entrevista telefónica		89,2 (82,1–93,7)	99,7 (99,3–100)	0,92 (0,88–0,97)		0,89
Dong Z *et al*. (China, 2014) [28]	Sistema informático de diagnóstico de cefaleas		99,4 (96,5–99,9)	97,9 (95,9–98,9)	0,96 (0,88–1,00)		0,97
Sasaki S *et al*. (Japón, 2023) [29]	Modelo de IA para el diagnóstico de migraña en pediatría	94,5	88,7	96,5	0,86 (0,77–0,91)	0,86	

*CefaleApp* muestra un rendimiento muy competitivo, especialmente en sensibilidad (97%) y F1-score (0,95) en población adulta (migraña vs no migraña). Su especificidad es algo menor (73%) de la que muestran otras herramientas digitales, pero está en línea con estudios que incorporan población más general o síntomas menos típicos. El valor de Kappa de 0,75 es ligeramente inferior a las mejores herramientas (κ ~ 0,87–0,90), pero sigue siendo un acuerdo sustancial-alto, y el MCC de 0,73 confirma un rendimiento equilibrado. Dado que *CefaleApp* está diseñada específicamente para diagnosticar migraña vs cefalea tipo tensión, sus valores son adecuados y muestran robustez para el ámbito clínico de Atención Primaria.

La especificidad moderada en el diagnóstico de migraña indica una ligera tendencia a sobre diagnosticar esta entidad en pacientes sin migraña. Así, el 19% de los pacientes con cefalea tipo tensión son clasificados por *CefaleApp* como pacientes con migraña, y en el 41,7% de los pacientes diagnosticados con cefalea mixta, los casos fueron clasificados como personas con migraña por *CefaleApp*, mientras que el neurólogo los clasificó como una cefalea de características mixtas con un “predominio de migraña”. Esto último indica que, en los pacientes clasificados como una cefalea mixta por el neurólogo, las cefaleas tipo migraña fueron correctamente identificadas por la app. Sin embargo, esta reclasificación no tuvo un impacto relevante en su capacidad discriminativa global, reflejada en un índice de Youden de 0,71 para el diagnóstico de migraña.

El algoritmo de diagnóstico de *CefaleApp* se basa en los criterios diagnósticos de la ICHD-III, y esta clasificación no reconoce la categoría de cefalea mixta, aunque la cefalea tipo tensión está incluida en la categoría de migraña crónica, y en el caso de combinar cefalea tipo tensión y migraña probable, el diagnóstico sería el de cefalea tipo tensión [22]. La práctica clínica real no se basa sólo en el empleo de unos criterios diagnósticos, y emitir el juicio clínico de que una persona tiene una “cefalea de características mixtas” no es excepcional. Normalmente, esta situación se suele corresponder con pacientes que tienen cefaleas crónicas y que suelen llevar asociadas las comorbilidades de ansiedad y/o depresión. Estos pacientes son una realidad en las consultas de Neurología, y representan un reto para el neurólogo general, que es el principal consultor de personas con cefalea en la mayoría de las consultas de los hospitales.

Entre los posibles sesgos y limitaciones de la validación de *CefaleApp* se encuentran los siguientes: (1) en la selección de los pacientes falta la validación multicéntrica en la APS, aunque centrarse en perfiles comunes mitiga parte del sesgo, (2) el espectro de enfermedad supone un sesgo bajo ya que la app se dirige solo a las cefaleas primarias más frecuentes que son la migraña y la cefalea tipo tensión, mientras que las otras cefaleas se excluyen como criterio de aplicabilidad, (3) aunque el neurólogo es la única referencia (gold estándar), el sesgo es bajo ya que el diagnóstico de migraña o de cefalea tipo tensión es más estable entre clínicos al basarse en criterios clínicos bien definidos, (4) el sesgo de generalización existe ya que se necesita validar la aplicación en el ámbito de la APS, (5) la exclusión de casos no aplica como sesgo ya que la exclusión de otras cefaleas forma parte del diseño (la app no pretende diagnosticarlas), y (6) la interpretación retrospectiva es un sesgo menor, ya que al considerar las cefaleas mixtas como válidas es clínicamente coherente (capturan el perfil migrañoso o el tensional predominante en el paciente).

Como se comentó en el apartado de Material y Métodos, *CefaleApp* dispone de un algoritmo terapéutico en el que se describen los posibles tratamientos sintomáticos (AINEs, triptanes, diptanes, gepantes, ergóticos) y el tratamiento preventivo más adecuado teniendo en cuenta las diversas comorbilidades del paciente (Fig. 1). Así, en el caso de señalar que nuestro paciente con migraña tiene asma bronquial, litiasis renal e hipotensión arterial, *CefaleApp* excluirá de las recomendaciones del tratamiento preventivo a los betabloqueantes, los antihipertensivos (lisinopril, candesartán) y los neuromoduladores (topiramato, zonisamida), y señalará como indicados a la amitriptilina, la venlafaxina, la flunaricina, el ácido valproico y, aunque de uso hospitalario, también señalará la posibilidad de los diversos anticuerpos monoclonales anti-CGRP y los gepantes, así como de la toxina botulínica. Un mini-vademécum específico de estos fármacos aporta una sucinta información sobre posología, efectos adversos y contraindicaciones. Además, se incluye un apartado con las situaciones especiales de la gestación y de la lactancia, ayudando al profesional a seleccionar el tratamiento más adecuado. La información farmacológica se va actualizando periódicamente.

Los beneficios que se podrían esperar de una implementación generalizada de *CefaleApp* serían una disminución de la demora del tiempo hasta la confirmación del diagnóstico de migraña y una mejora en la adecuación terapéutica a las comorbilidades del paciente. El empoderamiento de la persona con migraña en el conocimiento de su cefalea se podría traducir en un aumento de la adherencia terapéutica y de la satisfacción de los pacientes. Finalmente, desde el punto de vista del sistema sociosanitario, *CefaleApp* también podría contribuir a la equidad asistencial, especialmente en zonas con menos recursos o baja especialización médica.

## 6. Conclusiones

*CefaleApp* es una herramienta digital innovadora cuyo desarrollo está basado en criterios clínicos validados, y que ha demostrado su utilidad en contextos clínicos reales. *CefaleApp* podría ser de utilidad para apoyar el diagnóstico de la migraña en la APS, lo que a su vez podría fomentar el uso del tratamiento sintomático con triptanes e iniciar de forma precoz el tratamiento preventivo considerando las comorbilidades del paciente.

## Data Availability

Los datos numéricos se incluyen en el manuscrito. *CefaleApp* es una app de descarga libre para dispositivos Android a través de Google Play Store. Para recibir información sobre el proceso de programación se puede contactar con el desarrollador de la aplicación a través del correo baltasarq@gmail.com.

## Contribuciones de los Autores

DAGE diseño, recopilación y análisis de datos, y escritura del manuscrito. BGPS diseño, programación de la app (Java), lectura crítica del manuscrito. NASC diseño, recopilación de datos y lectura crítica del manuscrito. Todos los autores contribuyeron a los cambios editoriales en el manuscrito. Todos los autores leyeron y aprobaron el manuscrito final. Todos los autores han participado lo suficiente en el trabajo y han acordado ser responsables de todos los aspectos del mismo.

## Aprobación Ética y Consentimiento Informado

El estudio dispone del informe favorable del Comité de Ética de la Investigación con medicamentos de Galicia con el código de registro 2024/528. Los pacientes que participaron en la validación de la app dieron su consentimiento informado por escrito. El estudio se llevó a cabo de acuerdo con las directrices de la Declaración de Helsinki.

## Financiación

Esta investigación no recibió financiación externa.
